# Mavorixafor, an Orally Bioavailable CXCR4 Antagonist, Increases Immune Cell Infiltration and Inflammatory Status of Tumor Microenvironment in Patients with Melanoma

**DOI:** 10.1158/2767-9764.CRC-22-0090

**Published:** 2022-08-31

**Authors:** Robert H.I. Andtbacka, Yan Wang, Robert H. Pierce, Jean S. Campbell, Melinda Yushak, Mohammed Milhem, Merrick Ross, Katie Niland, Robert D. Arbeit, Sudha Parasuraman, Kris Bickley, Cecilia CS Yeung, Lauri D. Aicher, Kimberly S. Smythe, Lu Gan

**Affiliations:** 1 Surgical Oncology, Huntsman Cancer Institute, University of Utah, Salt Lake City, Utah.; 2 X4 Pharmaceuticals, Boston, Massachusetts.; 3 Clinical Research Division, Fred Hutchinson Cancer Research Center, Seattle, Washington.; 4 Department of Hematology and Medical Oncology, Emory University School of Medicine, Atlanta, Georgia.; 5 Medical Oncology, University of Iowa, Iowa City, Iowa.; 6 Surgical Oncology, MD Anderson Cancer Center, University of Texas, Houston, Texas.

## Abstract

**Purpose:**

Mavorixafor is an oral, selective inhibitor of the CXCR4 chemokine receptor that modulates immune cell trafficking. A biomarker-driven phase Ib study (NCT02823405) was conducted in 16 patients with melanoma to investigate the hypothesis that mavorixafor favorably modulates immune cell profiles in the tumor microenvironment (TME) and to evaluate the safety of mavorixafor alone and in combination with pembrolizumab.

**Experimental Design:**

Serial biopsies of melanoma lesions were assessed after 3 weeks of mavorixafor monotherapy and after 6 weeks of combination treatment for immune cell markers by NanoString analysis for gene expression and by multiplexed immunofluorescent staining for *in situ* protein expression. Serum samples taken at biopsy timepoints were evaluated for key chemokine and cytokine alterations using the Myriad Rules Based Medicine multiplex immunoassays.

**Results:**

Within the TME, mavorixafor alone increased CD8^+^ T-cell infiltration, granzyme B signal, antigen presentation machinery, and both tumor inflammatory signature (TIS) and IFNγ gene expression signature scores. Increases in the key serum cytokines CXCL9 and CXCL10 were further enhanced when mavorixafor was combined with pembrolizumab. Adverse events (AE), as assessed by the investigator according to NCI Common Terminology Criteria for Adverse Events (v4.03), related to either mavorixafor or pembrolizumab (≥15%) were diarrhea, fatigue, maculopapular rash, and dry eye. Reported AEs were all ≤ grade 3.

**Conclusion/Discussion:**

Treatment with single-agent mavorixafor resulted in enhanced immune cell infiltration and activation in the TME, leading to increases in TIS and IFNγ gene signatures. Mavorixafor as a single agent, and in combination with pembrolizumab, has an acceptable safety profile. These data support further investigation of the use of mavorixafor for patients unresponsive to checkpoint inhibitors.

**Significance:**

Despite survival improvements in patients with melanoma treated with checkpoint inhibitor therapy, a significant unmet medical need exists for therapies that enhance effectiveness. We propose that mavorixafor sensitizes the melanoma tumor microenvironment and enhances the activity of checkpoint inhibitors, and thereby may translate to a promising treatment for broader patient populations.

## Introduction

Chemokine receptors mediate diverse cell migration processes in normal physiology and in disease through interactions with cognate ligands (1, 2). CXCR4 is one of the best-characterized chemokine receptors and performs essential functions in immune, neural, and hematopoietic stem cell (HSC) migration, inflammation, and human immunodeficiency virus (HIV) infection (3). In tissue, CXCR4 is expressed on a wide range of cell types, including normal stem cells, HSCs, mature lymphocytes, and fibroblasts (4). The soluble chemokine CXCL12 (previously referred to as SDF1α) is the sole ligand for CXCR4.

There is substantial evidence that the CXCR4/CXCL12 pathway also supports cancer development and survival by regulating angiogenesis and the trafficking of key immune cells in the tumor microenvironment (TME; ref. 5). Direct expression of one or both factors has been observed in a wide range of tumors (6–8), and expression of CXCR4/CXCL12 has been associated with a poor prognosis in multiple cancers, including breast, ovarian, renal, lung, and melanoma. CXCR4/CXCL12 expression has also been associated with increased risk of metastasis to lymph nodes, lung, liver and brain, all sites of CXCL12 expression (6, 9–11). As a result, CXCR4 and other chemokine receptors that promote tumor survival have increasingly become important targets for anticancer therapies (2).

In melanoma, CXCR4 is frequently expressed at increased levels, particularly in the CD133^+^ melanoma cell population believed to represent melanoma stem cells (12, 13). *In vitro* and murine model experiments have demonstrated that CXCL12 is chemotactic for CD133^+^ melanoma cells (14). In murine solid tumor models, including ovarian cancer, glioblastoma, and pancreatic cancer, the CXCR4 antagonist AMD3100 has been shown to decrease tumor angiogenesis (5, 15) and revascularization (16), reduce lung metastasis (17, 18), and induce infiltration of CD8^+^ T cells into tumors (15, 19). Moreover, treatment with CXCR4 antagonists has demonstrated robust inhibition of murine B16-OVA melanoma growth (20), and enhanced survival has been reported in multiple mouse models when a CXCR4 antagonist is combined with a checkpoint inhibitor (19, 20). Hence, inhibition of the CXCR4/CXCL12 pathway may represent a viable therapeutic pathway in melanoma.

Recently, the introduction of immunotherapies that target PD-1 and CTLA-4 checkpoint inhibition has dramatically improved the treatment of advanced malignancies, including advanced melanoma. Despite this, overall survival is still relatively poor for patients with advanced melanoma (21). Additional treatment options for advanced melanoma, both as a frontline therapy and in combination with checkpoint inhibitors, are required.

Mavorixafor is an oral, selective, allosteric CXCR4 inhibitor being developed for the treatment of Warts, Hypogammaglobulinemia, Infections, and Myelokathexis (WHIM) syndrome, melanoma, and other liquid and solid tumors (22–25). We hypothesized that disruption of CXCR4/CXCL12 signaling by mavorixafor would favor an improved response to checkpoint inhibitors by modulating the immune cell profile within the TME and increase CD8^+^ T-cell infiltration. The infiltration of CD8^+^ T cells into the TME is a critical determinant of effective immune responses to many types of solid tumors, including melanoma (26, 27). Here we report results from a biomarker-driven phase Ib clinical study in patients with melanoma to test this hypothesis (NCT02823405). The results suggest that mavorixafor enhances immune cell infiltration into melanoma metastases and may have the potential to improve clinical responses to checkpoint inhibitors.

## Materials and Methods

### Objectives

This was an open-label, phase Ib study to evaluate the safety and tolerability of mavorixafor alone and in combination with pembrolizumab in patients with advanced melanoma. In addition, the effects of mavorixafor monotherapy and combination treatment on melanoma lesion histology and inflammatory cell infiltrates were examined along with changes in serum biomarkers and peripheral blood mononuclear cell (PBMC) populations.

### Study Population

Study participants were ≥18 years of age with a histologically confirmed diagnosis of malignant melanoma with at least two separate cutaneous or subcutaneous lesions of at least 3 mm diameter that were suitable for punch biopsies. Exclusion criteria included prior treatment with immunotherapies, including anti-CTLA-4, PD-1, or PD-L1 checkpoint inhibitors; an Eastern Cooperative Oncology Group (ECOG) performance status ≥2; ongoing grade ≥2 adverse events (AE) according to NCI Common Terminology Criteria for Adverse Events (CTCAE v4.03) criteria; or uncontrolled infection, angina, congestive heart failure, diabetes, or seizures. In addition, patients presenting with screening laboratory values of hemoglobin <9.0 g/dL, creatine >2.0 × the upper limit of normal (ULN), serum aspartate transaminase, or alanine transaminase >3 × ULN, or absolute neutrophil counts or platelets <1,500/μL or 100,000/μL, respectively, were also excluded. All study participants provided written informed consent.

### Study Design

The study was conducted in accordance with the Declaration of Helsinki and International Conference on Harmonization guidelines for good clinical practice. The Institutional Review Board at each participating center approved the study protocol.

**FIGURE 1:**
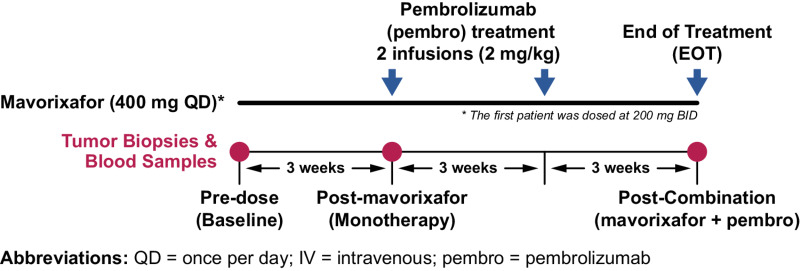
Study schematic. Sixteen patients received 400 mg mavorixafor every day for 9 weeks. Following 3 weeks of mavorixafor monotherapy, 6-week combination therapy was initiated by the addition of pembrolizumab (intravenous infusion). Sampling occurred at baseline, week 4 (post-monotherapy), and week 9 (post-combination therapy).

This was a phase Ib study of patients with advanced melanoma who were previously untreated with checkpoint inhibitor therapy. Sixteen patients received 400 mg mavorixafor administered orally once daily for 9 weeks. Following 3 weeks of initial mavorixafor monotherapy, combination therapy was initiated, with the addition of pembrolizumab administered as an intravenous infusion consistent with the prescribing information, that is, 2 mg/kg in 2 × 3-week cycles, at the week 4 and week 7 visits (Fig. 1). One patient began mavorixafor treatment with a 200 mg twice daily regimen under a prior protocol and maintained the twice daily dosing schedule throughout the study. The 400 mg daily dose of mavorixafor was chosen based on prior demonstration of pharmacologic activity, safety, and tolerability in previous studies with healthy volunteers (28, 29) and HIV-infected patients (30).

Blood samples for analysis of PBMCs and serum biomarkers, and serial biopsies of cutaneous and/or subcutaneous melanoma lesions were taken at baseline on day 1, at week 4 following the 3-week mavorixafor monotherapy period but prior to pembrolizumab dosing, and at week 9 at the end of the combination treatment period. The biopsy analyses focused primarily on post-mavorixafor monotherapy comparisons with baseline due to limited sample availability post-combination treatment.

### IHC

Single-marker IHC was performed on formalin-fixed paraffin-embedded (FFPE) tumor sample sections, (4 μm thickness) after heat-induced antigen retrieval (HIER, citrate buffer, pH 6.0), obtained from patients with melanoma at the designated treatment timepoints. Granzyme B was localized by immuno-labeling tissue sections with mouse mAb (clone GrB-7; DAKO) at 1:25 dilution followed by detection with the EnVisionTM G2 System/AP kit and visualization by Permanent Red Chromogen substrate staining. CD8 was labeled in FFPE tissue sections using mouse monoclonal anti-CD8 antibody (clone C8/144B; DAKO) at a 1:100 dilution followed by detection with the BOND Polymer Refine Red Detection Kit (Leica) and AP Red chromogen substrate visualization. Whole-slide scans were imaged using the Aperio-AT system (Leica Biosystems) and transferred into the HALO (Indica Labs) platform for quantitative digital image analysis.

### Multiplex Immunofluorescence

FFPE tumor samples were obtained from patients with melanoma at designated treatment timepoints. Slides were sequentially stained with antibodies after rounds of HIER (BOND Epitope Retrieval Solution 2, pH 9.0) and detected by antibody-binding horseradish peroxidas–containing polymers in conjunction with fluorescent-labeled tyramides (Opal, Akoya). DAPI was used as a nuclear stain. One 3-plex and three 6-plex multiplex antibody panels were optimized and applied to patient tissue samples (details in Supplementary Table ST1). Whole-slide scans were imaged using the Aperio-FL system (Leica Biosystems) or Vectra 3.0 (Akoya), and fluorochromes with spectral overlap were deconvolved and autofluorescence-subtracted (inForm software, Akoya). Images were analyzed using HALO software (Indica Labs). Cells were segmented on the basis of nuclear stain, and thresholds for antibody positivity were calibrated for each slide.

### NanoString Analysis

RNA was extracted from FFPE slides, from patients with biopsies with sufficient tissue at predose and mavorixafor monotherapy timepoints (*n* = 9), for NanoString analysis and analyzed using PanCancer Immune Profiling and PanCancer Progression Panels that were supplemented with 30 user-defined genes (NanoString Technologies). Raw counts were normalized using the geometric mean of housekeeping genes, and the normalized data from both panels were merged and scaled on the basis of the expression of 134 overlapping probes using nSolver software (Version 4.0). Signature scores were calculated by taking the log_10_ of the geometric mean of the normalized counts across predefined gene sets to generate specific gene expression scores. The CTL gene signature was calculated using the log_10_-transformed geometric mean of normalized counts for the gene set of *CD8A, CD8B, FLTLG, GZMM*, and *PRF1*. The tumor inflammatory signature (TIS) was similarly calculated from 18 genes, as described elsewhere (15). The antigen processing and/or presentation genes used to create the APP gene signature are available upon request. The IFNγ gene signature was determined using the gene set of *IFNγ, CXCL9, CXCL10, HLA-DRA, IDO1*, and *STAT1* (31). The granzyme B expression level was calculated using normalized raw granzyme B counts from patient samples.

### Serum Biomarker Measurement

Sera were prepared from collected blood samples and concentrations of chemokines, cytokines, and growth factors were measured using the Multi-Analyte Profile platform (MAP; Myriad RBM). The proteins examined include those in HCANCER2, HMP8, HMPC19, HMPC42 MAPs and IL15, IFNγ, and IL2 by Simoa (Quanterix).

### ImmunoSeq Data

To evaluate T-cell clonality, genomic DNA was isolated from paired FFPE samples and cryopreserved PBMCs using the Qiagen DNA extraction kit (QIAGEN), quantified by PicoGreen and Nanodrop per manufacturer instructions, and samples were submitted to Adaptive Biotechnologies for ImmunoSEQ hsTCRB v4 and v4b analyses according to Adaptive's guidelines. FASTA files were analyzed with the LymphoSeq R package (https://rdrr.io/bioc/LymphoSeq/) and only productive sequences were further analyzed. A productive sequence is defined as in-frame sequences that do not have an early stop codon. Only 3 patients had sufficient PBMC and pre/post-matched melanoma specimens for ImmunoSEQ analyses. Both complementarity determining region 3 nucleic acid and amino acid sequences were generated in addition to summary statistics. Specific functions within the LymphoSeq R package were utilized to create visualizations including repertoire diversity, commonSeqVenn, and commonSeq.

### Data Availability

Gene expression (Nanostring) and multispectral immunofluorescence (mIHC) data were generated by the authors and are available from the corresponding author upon request. ImmunoSeq data for this study were generated at Adaptive Biotechnologies. Derived data supporting the findings of this study are available from the corresponding author upon request.

## Results

### Patient Characteristics and Disposition

Sixteen patients received treatment between September 15, 2016 and March 15, 2018 at four study sites in the United States. A total of 10 males and 6 females were treated, with a median patient age of 74.5 years (Table 1).

**TABLE 1:** Participant demographic and baseline characteristics

Mavorixafor + Pembrolizumab (*N* = 16)		
Age (years)	Mean (± SD) Median (min, max)	74.6 (± 9.6) 74.5 (53, 91)
Gender	Male Female	10 (62.5%) 6 (37.5%)
Ethnicity	Not Hispanic or Latino	16 (100%)
Race	White Asian	15 (93.8%) 1 (6.3%)
Screening ECOG status	0 1	9 (56.3%) 7 (43.8%)
**Disease Characteristics (*N* = 16) *N* (%)**		
Resectable Disease	Yes No	10 (62.5) 6 (37.5)
Stage of Disease Enrollment^a^	IIIB IIIC IV M1A	4 (25.0) 10 (62.5) 2 (12.5)
Prior systemic therapies	0 1	15 (93.8) 1 (6.3)

Abbreviations: ECOG, Eastern Cooperative Oncology Group; SD, standard deviation.

^a^per AJCC 7th Edition.

Eligible patients had a screening ECOG performance status <2, and most (93.8%) had not received prior systemic therapy for their disease. Of the 16 study participants, 11 (68.8%) completed the study and 5 discontinued treatment, 4 (25%) because of AEs (further described in safety) and 1 (6.3%) who was lost to follow-up. The median time on treatment was 6.1 weeks (range, 3.0–10.0 weeks).

### Safety

Monotherapy with mavorixafor was generally well tolerated, with diarrhea (*n* = 5, 31%) and chills (*n* = 2, 12.5%) the only treatment-related AEs to occur in 2 or more patients (Table 2).

**TABLE 2:** AEs related to monotherapy and combination treatment^a^

(*N* = 16; >10% of Participants, All Grades and Grade 3)		
	All Grades, *N* (%)	Grade 3, *N* (%)
**Related to mavorixafor during 3-week monotherapy**		
All	11 (68.8%)	0
Diarrhea	5 (31.3%)	1 (6.3%)
Chills	2 (12.5%)	0
Fatigue	2 (12.5%)	0
**Related to mavorixafor or pembrolizumab during 9-week treatment**		
All	16 (100%)	0
Diarrhea	7 (43.8%)	1 (6.3%)
Fatigue	6 (37.5%)	0
Maculopapular rash	4 (25%)	2 (12.5%)
Dry eye	3 (18.8%)	0
Acute kidney injury	2 (12.5%)	1 (6.3%)
Chills	2 (12.5%)	0
Decreased appetite	2 (12.5%)	0
Dry mouth	2 (12.5%)	0
Ocular hyperemia	2 (12.5%)	0
Oral candidiasis	2 (12.5%)	0
Pruritis	2 (12.5%)	0
Rash	2 (12.5%)	0
Rash pruritic	2 (12.5%)	0

^a^Adverse events were assessed by the investigator according to NCI CTCAE (version 4.03).

There was only one grade 3 event related to mavorixafor during the 3-week monotherapy period (diarrhea in 1 patient) and no grade 4 or grade 5 events. Diarrhea (*n* = 7; 43.8%) was the most common AE related to either mavorixafor or pembrolizumab during the complete 9-week treatment period, followed by fatigue (*n* = 6; 37.5%), maculopapular rash (*n* = 4; 37.5%), and dry eye (*n* = 3; 25%). There were no grade 4 or 5 events related to combination treatment during the study, and maculopapular rash was the only related grade 3 event to occur in more than one patient during combination therapy (*n* = 2; 12.5%). AEs that led to treatment discontinuation by 4 study patients were single occurrences of diarrhea, stomatitis, palmar-plantar erythrodysesthesia syndrome, maculopapular rash, and immune-mediated adverse reaction. One patient experienced diarrhea during the initial mavorixafor monotherapy period that resolved upon discontinuation of study treatment. Events of stomatitis and immune-mediated adverse reaction were considered by the investigator to be related to pembrolizumab treatment. The events of palmar-plantar erythrodysesthesia syndrome and rash maculopapular were assessed by the investigator as related to both study drugs.

### Treatment with Mavorixafor Enhances Immune Cell Infiltration

**FIGURE 2:**
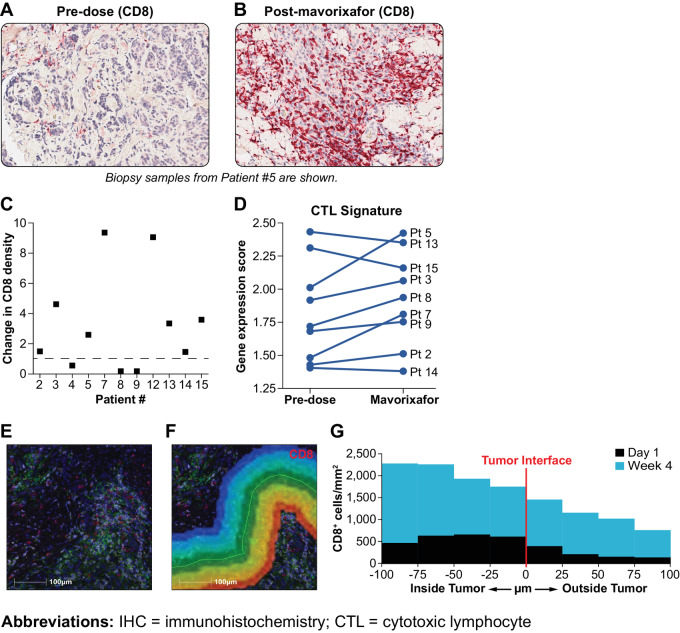
T-cell infiltration of the melanoma TME after mavorixafor therapy. Increased infiltration of the melanoma TME by CD8^+^ cells labeled by IHC predose (**A**) or at the end of mavorixafor monotherapy (**B**). Eight of 11 patient samples had a demonstrable increase in the fold change of CD8^+^ cell within the melanoma sample when compared before and after monotherapy (**C**). CTL signature scores for available pairs of patient biopsies following mavorixafor monotherapy (**D**). CD8^+^ T cells at the melanoma tumor interface with normal tissue were labeled using multiplex IHC (**E**). CD8^+^ cells/mm^2^ using HALO image analysis and plotted against distance from the tumor boundary in 25 μm bands (**F**). Labeled cells within 100 μm inside or outside of the tumor boundary with normal tissue were quantified (**G**).

Melanoma biopsy samples were examined for evidence of altered CTL trafficking following mavorixafor monotherapy. IHC labeling revealed a higher number of CD8^+^ cells infiltrating the TME at the end of the 3-week mavorixafor treatment compared with baseline (Fig. 2A–C). NanoString analysis of extracted RNA from biopsy samples showed elevated gene expression scores in 6 of 9 treated patients for a panel of CTL genes following the 3-week period of mavorixafor compared with predose levels (Fig. 2D). Data shown are from all evaluable patient samples available at the time of analyses. To examine the infiltration of CTLs into melanoma lesions in more detail, biopsy samples at the region of the tumor interface with normal tissue were stained using multiplex IHC and quantified for CD8^+^ cells by HALO analysis. After mavorixafor monotherapy, CD8^+^ cell density within the boundary area increased 4-fold compared with predose values (Fig. 2E–G).

### Mavorixafor-activated Immune Cell Activities in TME

**FIGURE 3:**
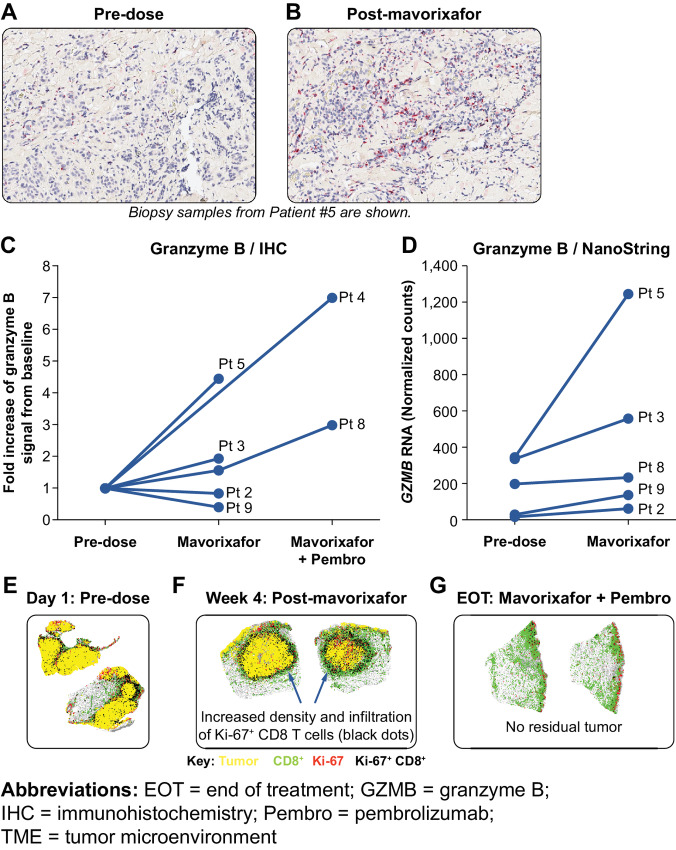
Mavorixafor-activated immune cell activities in TME. IHC labeling for granzyme B at predose (**A**) and postdose timepoints (**B**). The fold change of granzyme B positivity posttreatment for all evaluable samples (**C**). Quantification was performed using HALO software and the entire tumor area was scored. RNA expression levels for *GZMB* for 5 patients with both pre- and post-mavorixafor monotherapy treatment-evaluable biopsies (**D**). Data shown (**C** and **D**) is from all evaluable patient samples available at the time of analysis. Biopsies of melanoma lesions from patient #5 stained by mIF for CD8^+^ cells, Ki-67 (proliferating cells), and a cocktail of melanoma-specific antibodies to label tumor (**E--G**). Images represent the graphical output from the nearest-neighbor analysis module, with unlabeled cells rendered as gray.

Granzyme B produced from secretory granules by CTLs within tumor tissues is an important mediator of CD8^+^ T-cell effector function (32). At the end of the 3-week monotherapy period, IHC labeling indicated that mavorixafor increased granzyme B levels in some patient biopsy samples relative to baseline (Fig. 3A and B). Granzyme B expression increased further in available biopsies examined at the end of the combination therapy period (Fig. 3C). Elevated granzyme B protein levels following monotherapy correlated with increased *GZMB* RNA expression by NanoString analysis (Fig. 3C and D). The increase in granzyme B associated with cytolytic effector function is consistent with greater tumor infiltration by CD8^+^ T cells. A nearest-neighbor analysis was conducted on biopsy tissues stained for melanoma tissue antigens and CD8^+^ cells by multiplex immunofluorescence (mIF). After mavorixafor monotherapy, the average distance between CD8^+^ cells and the nearest tumor cell decreased from 95 μm at baseline to 43 μm. In addition, the number of unique neighbors increased, indicating enhanced infiltration by proliferating CTLs (Fig. 3E and F). This increase in TME infiltration by CD8^+^ cells at the end of the mavorixafor monotherapy period preceded the elimination of melanoma lesions observed with mavorixafor + pembrolizumab combination therapy (Fig. 3G).

### Mavorixafor Increases TIS and IFNγ Signature Scores and Expression of Antigen Presentation/Processing Genes

**FIGURE 4:**
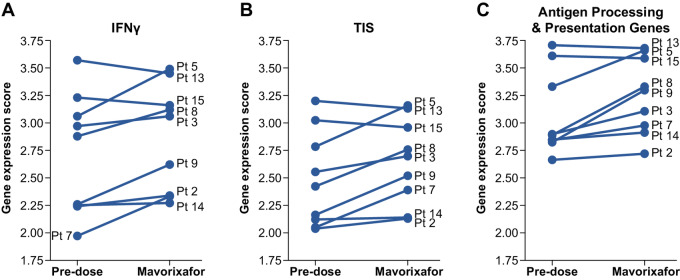
CXCR4 inhibitor affects the TME. Increased gene expression scores for IFNγ (**A**) and TIS (**B**) as determined by NanoString analysis of RNA extracted from patient biopsy samples at predose and post-mavorixafor timepoints. Gene expression scores for antigen processing and presentation genes as determined by NanoString analysis for 9 patients receiving Mavorixafor monotherapy (**C**). Data shown are from all evaluable patient samples available at the time of analyses.

The increased expression of genes for tumor cell epitope presentation to CTLs by mavorixafor was accompanied by similarly elevated tumor inflammatory gene signature (TIS) scores (Fig. 4A) and gene expression scores for IFNγ responses: *CXCL9, CXCL10, HLA-DRA, IDO1,* and *STAT1* (ref. 31; Fig. 4B), which are indicative of enhanced immune cell activation. Tumor cell killing by CTLs is enabled by T-cell receptor (TCR) recognition of target antigens presented by HLA class I (HLA-I)/beta-2-microglobin (B2M) complexes on target cells. The presentation of tumor-specific antigens activates effector mechanisms that directly target melanoma cells and activate additional antitumor responses. Peptide antigens are processed in intracellular proteasomes and translocated into the endoplasmic reticulum by the TAP1/TAP2 transport complex (33). At the end of 3-week mavorixafor monotherapy, expression scores for a panel of antigen processing and presentation genes measured in melanoma biopsies, which included *B2M*, *TAP1/TAP2*, and multiple *HLA* complex genes, trended toward an increase (Fig. 4C).

An analysis of serum protein changes after mavorixafor monotherapy using the MAP platform confirmed that several cytokines and chemokines were altered relative to baseline by mavorixafor monotherapy treatment (Supplementary Table ST2). In particular, serum CXCL9 (*P* = 0.0012) and CXCL10 (*P* = 0.0157) levels increased. These increases became substantially more robust after mavorixafor + pembrolizumab combination treatment, resulting in median fold increases of 5.23 (*P* < 0.0001) and 2.79 (*P* = 0.0004) for CXCL9 and CXCL10, respectively. These elevations in key serum cytokines are consistent with immune stimulation observed in other melanoma models (34), and together with increased IFNγ gene expression scores, support the use of mavorixafor in combination with checkpoint inhibitor therapies.

### Mavorixafor Expands T-cell Diversity in the TME

**FIGURE 5:**
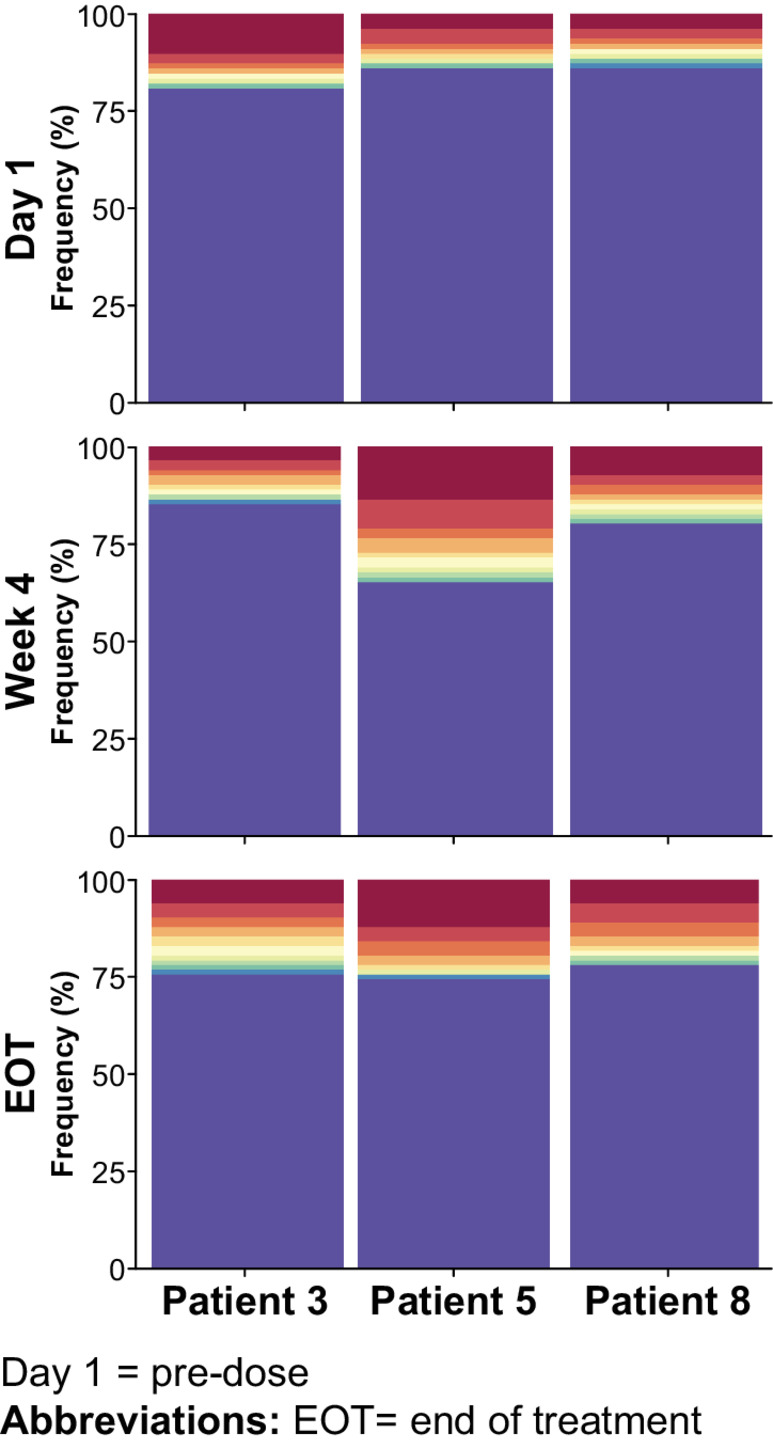
Repertoire diversity of patients 3, 5, and 8 demonstrating cumulative clonal frequency. Repertoire diversity obtained by plotting the cumulative frequency of a selected number of the most frequent clones using the function topSeqsPlot. Each of the top sequences is represented by its own color, with less-frequent clones assigned a single color (violet).

Tumor-infiltrating lymphocyte (TIL) repertoire diversity was analyzed in patients 3, 5, and 8 at baseline (day 1), week 4 (post-monotherapy), and end of treatment (EOT; post-combination therapy; Fig. 5). An expansion of T-cell repertoire diversity was readily observed in the tumor sample taken from patient 5 following monotherapy treatment (Table 3). After treatment with pembrolizumab, T-cell repertoire diversity diminished, suggesting that melanoma-specific T cells were expanding. In patient 3, the baseline tumor sample contained the highest percentage of clonal sequences. Patient 3 showed no significant clonal expansion between baseline and week 4, but clonal expansion of the top productive sequence was observed between week 4 and EOT. Patient 8 showed mild clonal expansion of top productive sequences between baseline and week 4 following monotherapy, with continued clonal expansion at EOT.

**TABLE 3:** Clonality of the TME of patients 3, 5, and 8

Patient	Timepoint	Total sequences	Unique productive sequences	Top productive sequence	Clonality^a^
**3**	D1	1589	1318	10.25	0.1127
	W4	3074	2608	3.6	0.0907
	EOT	400	334	6.84	0.0729
**5**	D1	3300	2668	4.05	0.0914
	W4	2289	1841	14.21	0.2211
	EOT	4626	3704	12.40	0.1590
**8**	D1	2562	2144	3.40	0.0990
	W4	601	513	6.85	0.0687
	EOT	2336	1983	5.87	0.1374

^a^Clonality is a representation of clonal diversity and ranges from 0 (all unique sequences) to 1 (all identical sequences). Patient #5 shows a dramatic increase in clonal diversity at week 4 following monotherapy, and though there was a decrease by EOT, clonal diversity remained substantially higher than at day 1. Patient #8 demonstrated an increase in clonal diversity at the end of study compared with day 1. Patient #3 did not show a concomitant increase in clonal diversity.

To further assess whether mavorixafor monotherapy expands intratumor T-cell diversity, we analyzed clonal sequences circulating in the blood and tumor of individual patients, as well as between patients. Top clonal sequences were identified and compared in the peripheral blood (PBMCs) and in the tumor. Few TCR sequences were shared by all patients (two sequences at day 1 and one sequence at EOT). Shared TCR TIL sequences (292) were identified at all three timepoints for patient 5 (Supplementary Fig. SF1). These sequences were found in the periphery (PBMCs), and monotherapy appeared to move T cells with identical sequences to the tumor at week 4. After treatment with pembrolizumab, these monotherapy-expanded specific sequences were detectable in the tumor or both the tumor and peripheral blood (Supplementary Fig. SF2).

## Discussion

This phase Ib study was conducted to determine the safety and tolerability of mavorixafor and pembrolizumab combination therapy and examine the ability of mavorixafor to favorably alter TME immune cell infiltration and biomarker responses to checkpoint inhibitor therapy via CXCR4 inhibition. Combination therapy was shown to be generally well tolerated in patients with advanced melanoma (stage IIIB–IV M1A; Table 1). Only one grade 3 event (diarrhea) was reported during the 3-week mavorixafor monotherapy period, which was also the only grade 3 event to occur in more than 1 patient (*n* = 2) during combination treatment. There were no grade 4 or 5 AEs related to either drug during the 9-week study period. These mavorixafor monotherapy safety results were consistent with previous findings that a daily dose of 400 mg mavorixafor was well tolerated in healthy volunteers (28, 29), HIV-infected patients (30), and patients with WHIM syndrome (23).

We have hypothesized that CXCR4 inhibition may enhance antitumor responses by stimulating trafficking of effector cells into the TME. The role of immunosuppressive mechanisms that inhibit tumor cell killing, including PD-L1 blockade, is now well established (27, 32, 35). The infiltration of the TME by CD8^+^ cells and the production of granzyme B and IFNγ have been correlated with decreased metastatic invasion and improved melanoma treatment responses (26, 36–38). In animal models, CXCR4 inhibition has been shown to increase immune responsiveness to solid tumors through induction of CD8^+^ T-cell responses that promote favorable CD8^+^/FoxP3^+^ T-cell ratios in the TME (15, 19, 39).

After 3 weeks of mavorixafor monotherapy in patients with advanced metastatic melanoma, infiltration of the TME by CD8^+^ cells was suggested by increases in a majority of treated-patient CTL signature gene expression scores and IHC labeling of patient biopsy samples. These increases were particularly prominent at the tumor interface with normal tissue, where the density of CD8^+^ cells was increased 4-fold relative to baseline samples by mavorixafor treatment alone. Increased infiltration of the tumor boundary by CD8^+^ cells has been associated with effective clinical responses to melanoma (27) and are consistent with favorable increases in CD8^+^/FoxP3^+^ ratios previously observed in melanoma biopsy samples (40). In a pancreatic adenocarcinoma slice culture system, the combination of CXCR4 inhibition and PD-1 blockade led to redistribution of CD8^+^ T cells from the stroma into the tumor, T-cell proliferation, clonal T-cell expansion, and cancer cell death (41). TCR sequencing data from our patient population support this observation, with TIL expansion in patient 5 at weeks 4 and persistence of TIL clones in the residual tumor site after extinction of the melanoma at the end of study. A similar but less dramatic TIL clonal expansion was seen in the TCR sequencing data for patient 8.

A nearest-neighbor analysis of patient biopsy samples also confirmed that mavorixafor monotherapy substantially decreased the distance between CD8^+^ T cells and tumor cells in the TME. This close juxtaposition of CTLs and target cells promotes the cell–cell contact required for TCR recognition of MHC class-I antigens presented on the tumor cell surfaces, and subsequent target cell killing (38). We observed a clear increase in gene expression scores for proteins involved in class-I antigen processing and presentation (32, 33). This increase was accompanied by an accumulation of *GZMB* RNA expression and granzyme B protein, which directly mediates cancer cell cytolytic killing by CTL cells, in patient biopsy samples.

Moreover, IFNγ and TIS gene expression scores were also increased following mavorixafor monotherapy. These suggest the promotion of antitumor activity by additional immune effector cells (32), which could augment responsiveness to checkpoint inhibitors. In this regard, we detected a number of significant changes in serum cytokine and chemokine levels at the end of combination therapy. The IFNγ-inducible chemokines CXCL9 and CXCL10 were increased a median of 5.23- and 2.79-fold, respectively, at the end of the 9-week combination treatment period. Elevated expression of these chemokine receptors in solid tumors promotes CD8^+^ T-cell responses and is associated with positive overall responses in patients with metastatic melanoma (42, 43).

These results show that mavorixafor has potent effects as a monotherapy agent for increasing the recruitment of CD8^+^ cells to melanoma lesions, leading to tumor cell interactions, particularly within the TME. This enhancement in CTL trafficking, in combination with increased class-I HLA expression within the TME, promotes effector–tumor cell interactions that increase granzyme B, IFNγ, and TIS gene expression. We examined whether there was a relationship between mavorixafor-induced changes in the TME and *CXCR4* expression and did not observe a correlation between changes in CD8 density and CXCR4 gene expression. When anti-CXCR4 antibodies become available commercially, it would be interesting to determine the spatial relationship of CXCR4-specific cells in the TME. In addition, mavorixafor + pembrolizumab combination therapy favorably alters serum chemokine and cytokine levels and promotes melanoma lesion regression.

The current landscape of CXCR4 antagonists spans a wide therapeutic range. Parentally administered, plerixafor, in combination with GCSF is currently available for stem cell mobilization in patients with non–Hodgkin lymphoma or multiple myeloma. Motixafortide is under investigation for treatment of patients with multiple myeloma as well as stem cell mobilization. Although Balixafortide recently failed its phase III trial in breast cancer, further development of the compound is under investigation as combination therapy in earlier stage cancers. Mavorixafor is being studied in patients with WHIM, Waldenstrom's macroglobulinemia, and severe chronic neutropenia. In addition, Ad-214 is currently in preclinical work in fibrotic diseases and BL-8040 is in clinical development in leukemia.

Although the biomarker-driven design of this study does not allow for assessment of clinical and radiologic response to mavorixafor in this patient population, it would be interesting to explore this aspect in future research. Despite this limitation, the positive alterations in biomarker profiles support further investigation of the use of mavorixafor in combination with anti-PD-1 therapy for treatment of melanoma and other solid tumors.

## Supplementary Material

Supplementary Figure 1 Shared TCR TIL sequences taken from tumor biopsy of patient #5 at various timepoints.

Supplementary Figure 2 Depiction of T cell clone mobilization from peripheral blood to melanoma site on day 1 and at EOT.

Supplementary Table 1 Antibody panels used for mIF analyses.

Supplementary Table 2 Serum cytokine and chemokine changes at the end of the 3-week monotherapy period compared to baseline.
