# Supplementary material for: Mavorixafor, an Orally Bioavailable CXCR4 Antagonist, Increases Immune Cell Infiltration and Inflammatory Status of Tumor Microenvironment in Patients with Melanoma
Source: Cancer Res Commun. 2022 Aug 31;2(8):904–13. doi: 10.1158/2767-9764.CRC-22-0090 (PMC10010370; doi:10.1158/2767-9764.CRC-22-0090)
Supplement: Supplementary Figure 1 — Shared TCR TIL sequences taken from tumor biopsy of patient #5 at various timepoints. [file crc-22-0090-s01.pdf]

Supplemental Figure SF1: Shared TCR TIL Sequences for Patient #5

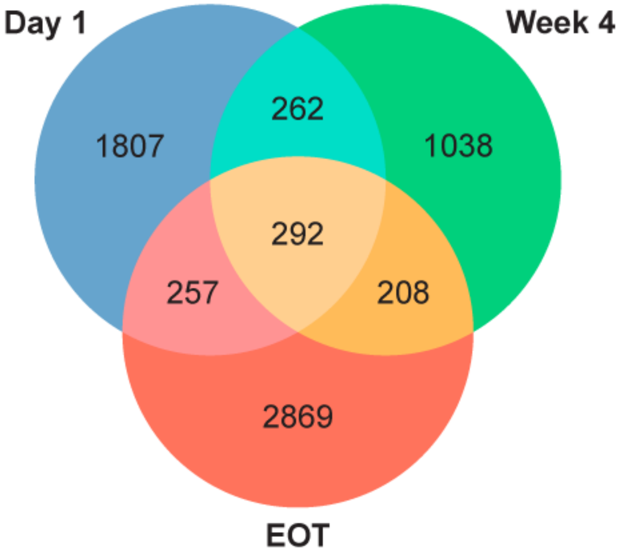

Abbreviations: EOT = end of treatment; TCR = T-cell receptor; TIL = tumor-infiltrating lymphocyte

The Venn diagram was created using the CommonSeqsVenn function to compare Day 1 (blue circle), Week 4 (green circle), and EOT (red circle) melanoma tissues biopsies taken from Patient #5. Of note, at the end of study no residual tumor was present and the entire residual tumor biopsy site (residual tumor bed) was subjected to TCR sequencing. 262 TIL sequences are shared between Day 1 and Week 4; 208 are shared between Week 4 and EOT; and 292 are shared between all three time points.
