# Supplementary material for: Mavorixafor, an Orally Bioavailable CXCR4 Antagonist, Increases Immune Cell Infiltration and Inflammatory Status of Tumor Microenvironment in Patients with Melanoma
Source: Cancer Res Commun. 2022 Aug 31;2(8):904–13. doi: 10.1158/2767-9764.CRC-22-0090 (PMC10010370; doi:10.1158/2767-9764.CRC-22-0090)
Supplement: Supplementary Figure 2 — Depiction of T cell clone mobilization from peripheral blood to melanoma site on day 1 and at EOT. [file crc-22-0090-s02.pdf]

Supplemental Figure SF2: T Cell Clone Mobilization from Peripheral Blood to Melanoma

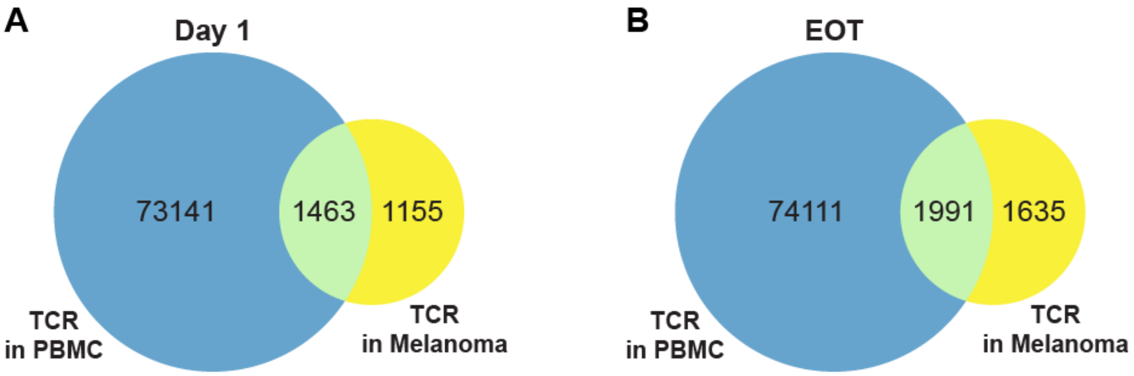

Abbreviations: TCR = T-cell receptor; PBMC = peripheral blood mononuclear cells; EOT = end of treatment

Blue circles depict TCR sequences seen in the PBMC sample. Yellow circles depict the TCR sequences seen in the melanoma tissue. The pale green area shows similar sequences between PBMC and melanoma. Panel A shows a comparison of TCR sequences at Day 1. Panel B shows a comparison of TCR sequences at EOT.
