# Supplementary material for: Mavorixafor, an Orally Bioavailable CXCR4 Antagonist, Increases Immune Cell Infiltration and Inflammatory Status of Tumor Microenvironment in Patients with Melanoma
Source: Cancer Res Commun. 2022 Aug 31;2(8):904–13. doi: 10.1158/2767-9764.CRC-22-0090 (PMC10010370; doi:10.1158/2767-9764.CRC-22-0090)
Supplement: Supplementary Table 1 — Antibody panels used for mIF analyses. [file crc-22-0090-s03.pdf]

Supplemental Table ST1: mIF Antibody Panel Configurations

3Mel_2 Panel					
Position	Antibody	Clone / Host	Company / Item	Concentration	OPAL Fluor
1	CD8	C8/144B / Mouse	DAKO / M7103	0.16 ug/ml	520
2	Melanoma Cocktail	M2-7C10; M2-9E3, T311, HMB45 / Mouse	Novus / NBP2-34337	0.03 ug/ml	650
3	Ki67	MIB-1 / Mouse	DAKO / M7240	0.23 ug/ml	570

AIR-5 Panel					
Position	Antibody	Clone / Host	Company / Item	Concentration	OPAL Fluor
1	CD8	C8/144B / Mouse	DAKO / M7103	0.05 ug/ml	520
2	CD163 & CD68	EP324 / Rabbit<br>PG-M1 / Mouse	Bio SB / BSB 3276<br>DAKO / M0876	0.125 ug/ml<br>0.04 ug/ml	650
3	PD-1	EH33 / Mouse	Cell Signaling / 43248	1:800	570
4	PD-L1	E1L3N / Rabbit	Cell Signaling / 13684	2.2 ug/ml	620
5	CD4	EP204 / Rabbit	Epitomics / AC0173A	0.08 ug/ml	690
6	FoxP3	236A/E7 / Mouse	eBioscience / 14-4777-82	5 ug/ml	540

SOX10_4					
Position	Antibody	Clone / Host	Company / Item	Concentration	OPAL Fluor
1	CD4	EP204 / Rabbit	Epitomics / AC0173A	0.08 ug/ml	690
2	CD8	C8/144B / Mouse	DAKO / M7103	0.2 ug/ml	520
3	PD-1	EH33 / Mouse	Cell Signaling / 43248	1:800	570
4	PD-L1	E1L3N / Rabbit	Cell Signaling / 13684	2.2 ug/ml	620
5	FoxP3	236A/E7 / Mouse	eBioscience / 14-4777-82	5 ug/ml	540
6	Melanoma Cocktail & Sox10	M2-7C10; M2-9E3, T311, HMB45 / Mouse & polyclonal / Rabbit	Novus / NBP2-34337<br>Cell Marque / 383A-74	0.05ug/ml<br>0.75ug/ml	650

VISTA_2					
Position	Antibody	Clone / Host	Company / Item	Concentration	OPAL Fluor
1	CD3	SP7 / Rabbit	Thermo / RM-9107	0.06 ug/ml	520
2	COX-2	RBT-COX2 / Rabbit	Bio SB / BSB 5362	0.5 ug/ml	540
3	B7H3/CD276	RBT B7H3 / Rabbit	Bio SB / BSB 2814	1 ug/ml	650
4	VISTA	D1L2G / Rabbit	Cell Signaling / 64953S	(1:100)	620
5	CD206	CLO387 / Mouse	Novus / NBP2-52927	1 ug/ml	570
6	CD163	EP324 / Rabbit	Bio SB/ BSB 3276	0.06 ug/ml	690
