# Supplementary material for: Mavorixafor, an Orally Bioavailable CXCR4 Antagonist, Increases Immune Cell Infiltration and Inflammatory Status of Tumor Microenvironment in Patients with Melanoma
Source: Cancer Res Commun. 2022 Aug 31;2(8):904–13. doi: 10.1158/2767-9764.CRC-22-0090 (PMC10010370; doi:10.1158/2767-9764.CRC-22-0090)
Supplement: Supplementary Table 2 — Serum cytokine and chemokine changes at the end of the 3-week monotherapy period compared to baseline. [file crc-22-0090-s04.pdf]

Supplemental Table ST2: Biomarker Changes Following Mavorixafor Monotherapy

Biomarker	Signed Rank	Student's t
**Increase**		
TNF-Related Apoptosis-Inducing Ligand Receptor 3 (TRAIL-R3)	< 0.0001	< 0.0001
Interleukin-6 receptor (IL-6r)	0.0002	0.0007
Myeloid Progenitor Inhibitory Factor 1 (MPIF-1)	0.0002	< 0.0001
Tumor necrosis factor receptor 2 (TNFR2)	0.0004	0.0005
Interleukin-2 Simoa (IL-2 Simoa)	0.0006	0.0063
Monokine Induced by IFN-gamma (MIG; CXCL9)	0.0012	0.0194
EN-RAGE	0.0020	0.0041
Tumor Necrosis Factor Receptor I (TNF RI)	0.0021	0.0032
Eotaxin-2	0.0026	0.1533
Chemokine CC-4 (HCC-4)	0.0034	0.0006
Urokinase-type plasminogen activator receptor (uPAR)	0.0034	0.0029
Interleukin-2 receptor alpha (IL-2 receptor alpha)	0.0103	0.0073
Macrophage Inflammatory Protein-1 beta (MIP-1 beta)	0.0103	0.0264
IFN-gamma Induced Protein 10 (IP-10; CXCL10)	0.0157	0.1099
6Ckine	0.0210	0.1296
Macrophage inflammatory protein 3 beta (MIP-3 beta)	0.0353	0.0807
Macrophage-Derived Chemokine (MDC)	0.0353	0.0680
AXL Receptor Tyrosine Kinase (AXL)	0.0463	0.0555
Tissue Inhibitor of Metalloproteinases 1 (TIMP-1)	0.0616	0.0278
**Decrease**		
Plasminogen Activator Inhibitor 1 (PAI-1)	0.0007	0.0007
Brain-Derived Neurotrophic Factor (BDNF)	0.0081	0.0040
Epidermal Growth Factor (EGF)	0.0237	0.0302
E-Selectin	0.0327	0.3136
Monocyte Chemotactic Protein 2 (MCP-2)	0.0377	0.0289

Significant serum cytokine and chemokine changes at the end of the 3-week monotherapy period compared to baseline levels. Samples from all evaluable patient samples were analyzed. Expressed as nominal p values.
